# Accelerated one-step generation of full-color holographic videos using a color-tunable novel-look-up-table method for holographic three-dimensional television broadcasting

**DOI:** 10.1038/srep14056

**Published:** 2015-09-11

**Authors:** Seung-Cheol Kim, Xiao-Bin Dong, Eun-Soo Kim

**Affiliations:** 1 HoloDigilog Human Media Research Center (HoloDigilog), 3D Display Research Center (3DRC), Kwangwoon University, 447-1Wolge-Dong, Nowon-Gu, Seoul 139-701, Korea

## Abstract

A color-tunable novel-look-up-table (CT-NLUT) for fast one-step calculation of full-color computer-generated holograms is proposed. The proposed method is composed of four principal fringe patterns (PFPs) such as a baseline, a depth-compensating and two color-compensating PFPs. CGH patterns for one color are calculated by combined use of baseline-PFP and depth-compensating-PFP and from them, those for two other colors are generated by being multiplied by the corresponding color-compensating-PFPs. color-compensating-PFPs compensate for differences in the wavelength between two colors based on their unique achromatic thin-lens properties, enabling transformation of one-color CGH pattern into those for other colors. This color-conversion property of the proposed method enables simultaneous generation of full color-CGH patterns, resulting in a significant reduction of the full color-CGH calculation time. Experimental results with test scenario show that the full color-CGH calculation time of the proposed CT-NLUT has been reduced by 45.10%, compared to the conventional NLUT. It has been further reduced by 96.01% when a data compression algorithm, called temporal redundancy-based NLUT, was used together, which means 25-fold reduction of its full color-CGH calculation time. Successful computational and optical reconstructions of full color-CGH patterns confirm the feasibility of the proposed method.

Thus far, the holographic method has been regarded as an ultimate approach for realistic three-dimensional (3-D) display because it can create the most authentic illusion of observing 3-D objects in motion without use of any special glasses^1^,^2^,^3^,^4^. In addition, this holographic 3-D display has been considered as an alternative way to current stereoscopic displays having serious drawbacks of eye fatigue and dizziness, therefore numerous research works on holographic 3-D displays have been done for implementing a practical holographic three-dimensional television (3-DTV) system^4^,^5^,^6^,^7^. However, a challenging issue in holographic 3-DTV broadcasting is the huge amount of holographic video data to be transmitted over the channel. For solving this problem, two approaches have been exploited. One method is to generate holographic videos for the input 3-D object data and compress them at the transmitter. The compressed hologram data are then transmitted to the receiver^8^,^9^,^10^,^11^,^12^,^13^. The other method is to compress the input 3-D object data and transmit them to the receiver. Hologram data for the compressed 3-D object data are then locally generated at the receiver^14^,^15^,^16^,^17^,^18^.

Several algorithms to compress the hologram data have been proposed^8^,^9^,^10^,^11^,^12^,^13^, but hologram patterns are treated as random data due to weak correlations among hologram pixels unlike the object data being highly co-related. That is, the bandwidth burden of a channel expects to be considerably alleviated in case object data is transmitted instead of hologram data. Thus, the transmission of compressed 3-D object data over the network and generation of computer-generated holograms (CGHs) for them at the receiver, has been recognized as a reasonable approach for achieving a practical holographic 3-DTV system^14^,^15^,^16^,^17^,^18^. This local generation of CGH patterns at the receiver can also provide us with a useful flexibility to calculate hologram patterns which are well matched with the individual specification of the employed 3-D display system.

This approach, however, calls for real-time computation of the CGH patterns for display. For this, various CGH algorithms have been suggested, which include ray-tracing^2^,^3^, look-up-table (LUT)^19^,^20^, compressed look-up table (C-LUT)^21^, image hologram^22^, recurrence relation^23^,^24^,^25^, wave-front recording plane (WRP)^26^,^27^, double-step Fresnel diffraction (DSF)^28^, polygon^29^,^30^,^31^,^32^,^33^,^34^, novel-look-up-table (NLUT)^15^,^16^,^17^,^18^,^35^,^36^ and color-space conversion methods^37^. Moreover, for full-color holographic 3-DTV broadcasting, CGH patterns for each color of the red (*R*), green (*G*) and blue (*B*), must be generated at once in real-time. Since each color hologram is calculated depending on the wavelength of a light source, three separate calculation processes are required for generation of full-color CGH patterns^5^,^6^,^7^,^38^,^39^.

Thus, the total calculation time for the full-color hologram increases three times compared to that for the single-color hologram. Since this calculation time can be reduced just by accelerating the computational speed of each CGH algorithm, most research works in electro-holography have been focused on the development of fast CGH algorithms^15^,^16^,^17^,^18^,^40^,^41^,^42^. However, even though the CGH calculation time can be shortened using fast algorithms, those approaches must require three separate calculation processes for the generation of full-color CGH patterns. It means that the total full-color CGH calculation time is determined by the sum of the CGH calculation times taken for each of the *R*-, *G*- and *B*-color, which actually limits the computational speeds of the conventional CGH algorithms. Therefore, in this paper, we propose a color-tunable novel-look-up-table (CT-NLUT) method to simultaneously generate full-color CGH patterns in a single-step calculation process based on its unique achromatic thin-lens property. With this property, color conversions among three-color object data can be achieved, which allows a CGH pattern for one-color to be transformed into those for other colors. Therefore, the proposed method can generate three-color CGH patterns from a pre-calculated single-color CGH pattern without additional calculation processes, which results in a significant reduction of the full-color CGH calculation time.

The proposed CT-NLUT is composed of four principal fringe patterns (PFPs) such as a baseline, a depth-compensating and two color-compensating PFPs. In the proposed method, the CGH pattern for one color is calculated by combined use of baseline-PFP and depth-compensating-PFP, and those for two other colors are then generated from this pre-calculated CGH pattern just by being multiplied with corresponding color-compensating-PFPs. Here, two color-compensating-PFPs, acting just like color-conversion filters, compensate differences in wavelength between two colors. Moreover, baseline-PFP and depth-compensating-PFP represent the 1^st^ depth layer of the input 3-D video frame, and the PFP to compensate depth differences between the baseline and other depth layers, respectively. These PFPs are two components of the compressed NLUT(C-NLUT) algorithm, known as an accelerated version of the NLUT^43^. To confirm the feasibility of the proposed method, experiments are performed with animated 3-D video scenario, where each video frame is composed of full-color object points of 200,000. The results are then compared to those of the conventional methods in terms of the number of calculated object points, the calculation time per one object-point and the memory capacity.

In the NLUT method, principal fringe patterns for each depth layer of a 3-D object are calculated as forms of Fresnel zone plates (FZPs). Thus, PFPs can be treated as thin-lenses with their own focal lengths^35^,^43^ and each PFP shows a chromatic aberration just like the optical lens system. If a PFP for the *R*-color is reconstructed with the *R*-color, the corresponding object point is focused on its own depth. However, in case of being reconstructed with the *G*-color, the focused depth of the corresponding object point moves to a new location due to its chromatic aberration. However, this chromatic aberration of a PFP can be corrected by combined use of the corresponding color-compensating-PFP just like an optical achromatic doublet lens. That is, the PFP for the red color can be transformed into that for the green color just by being attached with the color-compensating-PFP for the *G*-color, which means compensation of the depth difference of the reconstructed object point. In other words, by being multiplied with proper color-compensating-PFPs, the PFP calculated for one color can be converted into those for other colors.

In addition, unlike other approaches, the NLUT method generates the CGH patterns of 3-D scenes based on a two-step process^15^,^16^,^17^,^18^,^40^,^41^,^42^. In the first step, which is referred to the preprocessing for video data compression, the number of object points to be calculated is minimized by removing as much of redundant object data between the consecutive 3-D video frames as possible using data compression algorithms based on its another unique property of shift-invariance^41^,^42^. In the following step, which is referred to as the main-processing for CGH calculation, hologram patterns for those compressed video data are calculated using the NLUT^15^,^16^,^17^,^18^,^40^. Thus, the computational speed of the NLUT can be enhanced not only by reducing the number of object points to be calculated, but also by reducing the CGH calculation time itself.

## Results

30 video frames for test 3-D video scenario which an airplane flying over the clouds in the sky are generated using the 3DS MAX. In the experiments, CGH patterns for the *R*-color are initially calculated, and color-compensation processes are then applied to them to generate two other CGH patterns for the *G-* & *B*-color. That is, these *G*- & *B*-color CGH patterns are generated from the pre-calculated *R*-color CGH patterns just by being multiplied with their respective color-compensating-PFPs, compensating differences in wavelength between the *R* and *G*, and the *R* and *B*, respectively.

**Figure 1:**
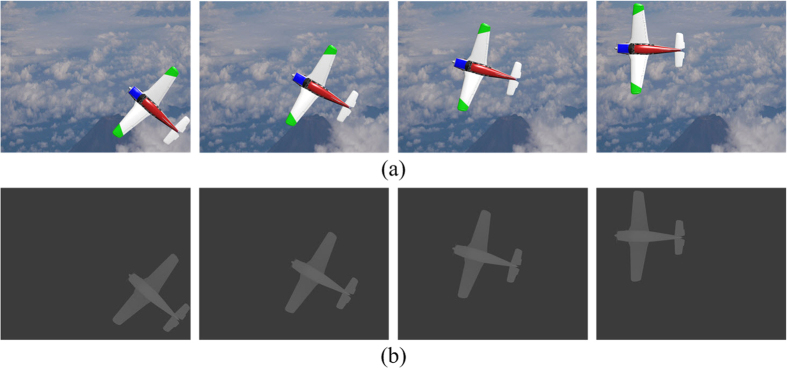
Intensity and depth images of the 1st, 10th, 20th and 30th frames for the animated ‘Airplane’ 3-D video scenario (**a**) intensity images, (**b**) depth images S.C.K. prepared intensity and depth data. S.C.K. took the background image.

Figure 1 shows intensity and depth images of the 1^st^, 10^th^, 20^th^ and 30^th^ frames for test scenario. Here, the resolution of 3-D video frames of test scenario is assumed to be 500 × 400 × 256 pixels, and each CGH pattern to be generated is assumed to have the resolution of 2,000 × 2,000 pixels, in which each pixel size is given by 10 *μm *× 10 *μm*. The horizontal and vertical discretization steps of less than 30 *μm* (100 *mm *× 0.003 = 30 *μm*) are chosen since the viewing-distance is assumed to be 100 *mm* here. Thus, to fully display fringe patterns, the PFP must be shifted by 1,500 pixels (500 × 3 pixels = 1,500 pixels) horizontally, and 1,200 pixels (400 × 3 pixels = 1,200 pixels) vertically, and the total resolution of the 2-D PFP becomes 3,500 (2,000 + 1,500) × 3,200 (2,000 + 1,200) pixels^35^. However, in the proposed CT-NLUT method, the 1-D sub-PFP is used and its resolution becomes 3,500 (2,000 + 1,500) × 1 pixels^44^.

### Performance analysis of the proposed method

As mentioned above, the total calculation time required for generation of full-color CGH patterns can be shortened by three consecutive processes of video data compression, single-color CGH and three-color CGH pattern calculations. The proposed CT-NLUT method, which is composed of four PFPs of a baseline-PFP, a depth-compensating-PFP and two color-compensating-PFPs, can calculate the single-color CGH pattern using the fast C-NLUT algorithm^43^, as well as directly generate three-color CGH patterns from this pre-calculated single-color CGH pattern. It means that the computational performance of the proposed method has been optimized in those processes of single and three-color CGH calculations, and its computational performance can be further enhanced in case a video data compression algorithm is employed.

For comparative performance analysis of the proposed method with those of the conventional methods, experiments are carried out for two cases with or without using a video data compression algorithm. In the first case, performances of the proposed CT-NLUT method are comparatively analyzed with those of the conventional NLUT method without using a video data compression algorithm. In the second case, those experiments are carried out under the condition that a video data compression algorithm called temporal redundancy-based NLUT (TR-NLUT) is employed. In the first case, the conventional NLUT method separately calculates CGH patterns for each color. But, in the proposed CT-NLUT method, only the *R*-color CGH pattern is calculated using the fast C-NLUT algorithm^43^, and *G*- & *B*-color CGH patterns are then generated from this calculated *R*-color CGH pattern by being multiplied with their respective color-compensating-PFPs.

Table 1 shows the average number of calculated object points of the conventional NLUT and proposed CT-NLUT methods for the first case. For generation of three-color CGH patterns, the conventional method performs CGH calculation operations for 600,000 (200,000 × 3) object points. In other words, 600,000 times of multiplication, shifting and adding processes are carried out in the conventional method^35^ In the proposed method, however, input object points are divided into three groups according to *R*, *G* and *B* grey levels of each object point. As seen in Table 1, 8,478, 9,169 and 182,353 object points are found to have the same grey levels in three, two and one colors, respectively.

The proposed method calculates the *R*-color CGH patterns at first. *G*- and *B*-color CGH patterns are then generated by two color-compensation processes for the object points having the same grey levels in all color components, one color- and one grey level-compensation processes for the object points having the same grey levels in two color components, and two color- and two grey level-compensation processes for the object points having the different grey levels in all color components.

The total number of CGH calculation operations of the proposed method for generating three-color CGH patterns becomes 574,131, which is the sum of 200,000, 256 and 373,875 (9,169 + 182,353 × 2) operations for generation of the *R*-color CGH pattern, color-compensation and grey level-compensation, respectively. That is, the numbers of CGH calculation operations of the proposed method have been reduced, compared to those of the conventional method. Moreover, as discussed in the chapter of ‘Method’, color-compensation is operated on the depth-by-depth basis, which means color-compensation operations can be done for all object points on the same depth at once simply by being multiplied with respective color-compensating-PFPs. Grey level-compensation operations are rather done on the point-by-point basis, but these are also simple multiplication processes unlike the conventional method requiring a series of multiplication, shifting and adding processes. This computational superiority of the proposed method over the conventional method both in calculations of single and three-color CGH patterns, allow a great reduction of the overall full-color CGH calculation time.

Table 2 show comparison results on the average calculation time per one object-point values of the NLUT and CT-NLUT methods, which are estimated to be 60.67 *ms* and 42.06 *ms*, respectively. It means that the proposed method has obtained 30.67% reduction of the calculation time per one object-point, compared to those of the conventional method. As the number of object points having different grey levels in all colors decreases, corresponding numbers of color- and grey-level compensation processes decreases correspondingly, which results in a great reduction of the CGH calculation time.

Now, the calculation time per one object-point of the proposed method can be further reduced just by decreasing the number of object points to be calculated for the single and three-color CGH patterns using one of the video data compression algorithms^15^,^16^,^17^,^18^,^40^. Thus, in the second case, the TR-NLUT algorithm^15^ is employed in both NLUT and CT-NLUT methods, which are called TR/NLUT and TR/CT-NLUT, respectively. That is, input 3-D video data are compressed with the TR-NLUT algorithm, and then single and three-color CGH patterns for those compressed object points are calculated with each of the NLUT and CT-NLUT methods. Unlike the first case, the proposed TR/CT-NLUT calculates the three-color CGH patterns only for the compressed video data, so that its computational performance expects to be further enhanced. Here, the TR/CT-NLUT method looks optimized in all three processes of video data compression, single and three-color CGH calculations. For the performance comparison, three-color CGH patterns for the compressed object data are also calculated using the conventional TR/NLUT method.

Table 1 also shows the average number of calculated object points of the TR/NLUT and TR/CT-NLUT methods. As seen in Table 1, the number of calculated object point values has been reduced down to 39,262, from 200,000 of the first case. In other words, input 3-D video data have been reduced by 80.37%, for the test scenario, compared to those of the first case. It means that 80.37% reduction of the number of calculated object point value, on the average, has been achieved in the second case by employing the TR-LUT algorithm. Thus, computational loads of the proposed method in the following calculation processes for single and three-color CGH patterns expect to be much alleviated.

In the conventional TR/NLUT method, three-color CGH patterns are separately calculated for all those compressed object points of 39,262, for test scenario. For generation of three-color CGH patterns, the conventional TR/NLUT must perform 117,786 (39,262 × 3) calculation operations. As seen in Table 1, object points of 39,262 are composed of object points of 15,793, 4,814 and 18,655 having the same grey levels in three, two and one colors. Thus, in the proposed TR/CT-NLUT method, *R*-color CGH patterns are initially calculated for 39,262 object points, *G*- & *B*-color CGH patterns are then generated from these *R*-color CGH patterns by performing color- and grey level-compensation operations to their respective *R*-color CGH patterns. Thus, the total numbers of CGH calculation operations of the TR/CT-NLUT become 81,642 (39,262 + 256 + 42,124 (=4814 + 18,655 × 2)), for test scenario. These results show that the numbers of CGH calculation operations of the TR/CT-NLUT of the second case have been significantly reduced by 85.78% compared to those of the CT-NLUT of the first case.

Table 2 shows comparison results on the average calculation time per one object-point of the TR/NLUT and TR/CT-NLUT methods. Those values are calculated to be 12.30 *ms* and 2.55 *ms*, respectively. It means that calculation time per one object-point values of the proposed TR/CT-NLUT have been found to be reduced by 79.27% compared to those of the conventional TR/NLUT method.

Table 3 shows detailed compositions of the calculation time per one object-point of the conventional NLUT, TR/NLUT and proposed CT-NLUT, TR/CT-NLUT methods. Here, the calculation time per one object-point are composed of the preprocessing time for video data compression and calculation times for generation of the *R*-color as well as both of the *G*- and *B*-color CGH patterns. As seen in Table 3, the total full-color CGH calculation time of the conventional NLUT has been calculated to be 60.67 *ms*, which is the sum of the *R*-color CGH calculation time of 20.22 *ms* and *G*- & *B*-color CGH calculation time of 40.45 *ms*. In other words, the total full-color CGH calculation time of the conventional NLUT equals three times of the *R*-color CGH calculation time.

The total full-color CGH calculation time of the CT-NLUT has been, however, reduced down to 42.06 *ms*, and it consists of 18.12 *ms* and 23.94 *ms* for the *R*-color CGH calculation and for both *G*- & *B*-color CGH calculations, respectively. Here, a reduction of the *R*-color CGH calculation time from 20.22 *ms* to18.12 *ms* has been resulted from the fact that the CT-NLUT method calculated the *R*-color CGH pattern using the fast C-NLUT algorithm. In addition, the CGH calculation time for both *G*- & *B*-color has been found to be much less than two times of that for the *R*-color. It is because the proposed method needs no other separate CGH calculation processes for both of the *G*- & *B*-color, instead it requires only one-step color- and grey level-compensation processes.

In the second experiment, the total full-color CGH calculation time of the TR/NLUT method has been estimated to be 12.30 *ms*, which is composed of three processing times such as 0.07 *μs* for the preprocessing, 4.10 *ms* for the *R*-color CGH calculation and 8.19 *ms* for *G*- & *B*-color CGH calculations. Among them, the preprocessing time looks too small to be ignored, compared to the CGH calculation time. Since the TR/NLUT calculates the CGH patterns only for the compressed object data, it’s *R*-color CGH calculation time has been reduced down to 4.10 *ms* from 20.22 *ms* of the conventional NLUT method. Thus, the *G*- & *B*-color CGH calculation time has been also calculated to be 8.19 *ms*, which is two times of the *R*-color CGH calculation time just like the case of the NLUT method.

On the other hand, in the proposed TR/CT-NLUT method, its total three-color CGH calculation time has been estimated to be the lowest value of 2.55 *ms*, which is composed of 0.07 *μs*, 1.56 *ms* and 0.99 *ms* for the preprocessing, *R*-color CGH calculation and *G*- & *B*-color CGH calculation, respectively. Here, the smallest CGH calculation times for the *R*-color as well as for the *G*- & *B*-color have been resulted from the fact that the fast C-NLUT algorithm is used for calculating the *R*-color CGH pattern only for the compressed object data, and simple one-step color- and grey level-compensation operations are employed for generation of the *G*- and *B*-color CGH patterns in the proposed TR/CT-NLUT method. In brief, the total full-color CGH calculation times of the proposed CT-NLUT and TR/CT-NLUT methods have been reduced down to 69.33% and 4.20%, respectively, on the average, compared to that of the conventional NLUT method.

**Figure 2:**
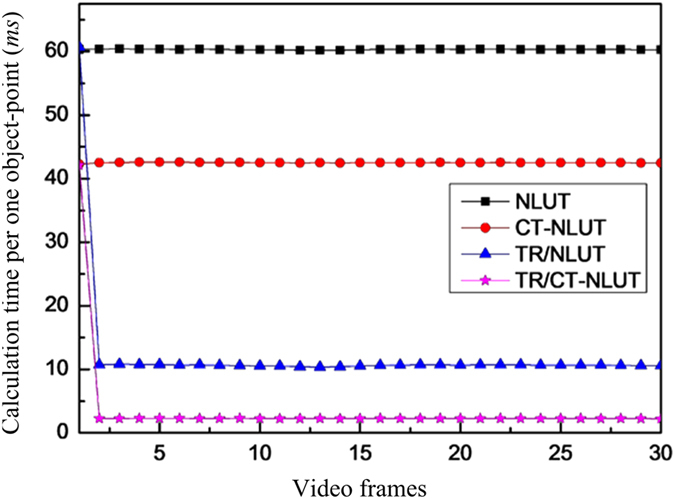
Comparison of the calculation time per one object-point of the NLUT, CT-NLUT, TR/NLUT and TR/CT-NLUT methods.

In addition, Fig. 2 shows the frame-based calculation time per one object-point variations of the conventional and proposed methods. As discussed above, calculation time per one object-point values of the CT-NLUT apparently appear to be much reduced, compared to those of the NLUT in all frames and scenarios.

As seen in Fig. 2, NLUT, TR/NLUT and CT-NLUT and TR/CT-NLUT methods, respectively, have the same calculation time per one object-point for the first frames. But, the first-frame calculation time per one object-point of the CT-NLUT and TR/CT-NLUT has been much reduced compared to those of the NLUT and TR-NLUT because full-color CGH patterns are calculated with the fast one-step process.

Table 2 also shows comparison results on the memory capacity required in the conventional and proposed methods. As seen in Table 2, the memory capacity of each of the conventional NLUT, TR/NLUT and proposed CT-NLUT, TR/CT-NLUT methods have been calculated to be 8.01 GB (3,500 × 3,200 × 8 bit × 768 = 8.01 GB) and 54.69 KB (3,500 × 1 × 8 bit × 8 = 54.69 KB), respectively, which means that 1.54 × 105-fold reduction of the memory capacity has been achieved in the proposed method, compared to that of the conventional method due to the fact that only eight 1-D sub PFPs are pre-calculated and stored in the proposed method^35^,^44^.

### Computational reconstruction of full-color CGH patterns

**Figure 3:**
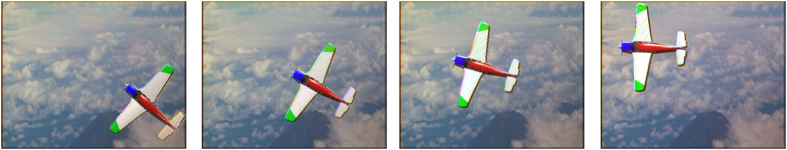
Computationally reconstructed 3-D video images for 1^st^, 10^th^, 20^th^ and 30^th^ frames. S.C.K. took the background image.

To confirm the feasibility of the proposed method in the practical application, 3-D video images have been computationally reconstructed from the full-color CGH patterns generated with the proposed TR/CT-NLUT method. Figure 3(a) shows computationally reconstructed 3-D images of the 1^st^, 10^th^, 20^th^ and 30^th^ video frames, respectively. All object images have been successfully reconstructed both in color and resolution.

## Conclusions

We proposed a new TR/CT-NLUT method, to fast calculate full-color holographic videos of 3-D scenes with a one-step calculation process based on its unique achromatic thin-lens property. Experimental results with test 3-D video scenarios show that the full-color CGH calculation time of the proposed TR/CT-NLUT method has been reduced by 95.58%, on the average, compared to that of the conventional NLUT method, which means 25-fold reduction of the full-color CGH calculation time. In addition, the memory capacity of the proposed method has been also reduced by 2.93 × 10^5^-fold compared to the conventional NLUT. Successful experimental results on the computational and optical reconstruction of full-color CGH patterns generated with the proposed method finally confirm the feasibility of the proposed method in the practical application fields.

## Methods

**Figure 4:**
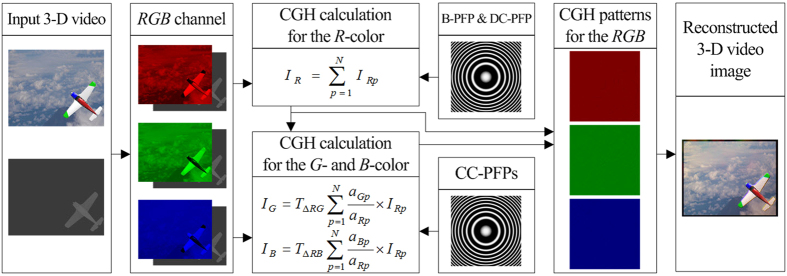
Overall functional-diagram of the proposed CT-NLUT method. S.C.K. took the background image.

Figure 4 shows an overall block-diagram of the proposed CT-NLUT method, which largely consists of four steps. First, the input full-color 3-D video frames are divided into three-color components (*R*, *G* and *B*). Second, the CGH pattern for the reference color (*R*) image is generated by combined use of baseline-PFP and depth-compensating-PFP. Third, CGH patterns for each of the *G*- & *B*-color are simultaneously generated just by multiplying the corresponding color-compensating-PFPs to the calculated *R*-color CGH pattern. Finally, full-color 3-D object images are reconstructed from these three-color CGH patterns generated with the proposed method. Of course, the input 3-D video data have to be practically compressed by using a data compression algorithm, and then input to the CGH calculation process of the proposed method of Fig. 4.

As mentioned above, in the NLUT method, a 3-D object is approximated as a set of discretely sliced depth image planes having different depth. Then, only the fringe patterns for the center-located object points on each depth plane, which are called PFPs, are pre-calculated and stored.

Therefore, the unity-magnitude PFP of the *R*-color (*T*_*R*_) for the object point (*x*_0_, *y*_0_, *z*_*p*_) which is positioned on the center of an image plane with a depth of *z*_*p*_, *T*_*R*_(*x*, *y*; *z*_*p*_), can be defined as Eq. (1)^35^


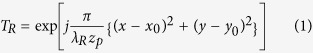


where *λ*_*R*_ represents the *R*-color wavelength. Here, if the wavelength difference between the *R* and *G*-color is set to be 1/Δ*λ*_*RG *_= 1/*λ*_*G*_ – 1/*λ*_*R*_, then the *G*-color PFP, *T*_*G*_ can be expressed by Eq. (2)


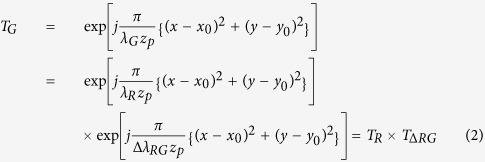


where *λ*_*G*_ denotes the *G*-color wavelength. Equation (2) reveals that the *G*-color PFP, *T*_*G*_ can be simply generated by multiplication of the PFP for the wavelength difference between the *R* and *G*-color, *T*_Δ*RG*_, which is called color-compensating-PFP for the *G*-color, to the *R*-color PFP, *T*_*R*_. Likewise, the *B*-color PFP, *T*_*B*_ can be generated by Eq. (3), where *T*_Δ*RB*_ represents the PFP for the wavelength difference between the *R*- and *B*-color, which is called color-compensating-PFP for the *B*-color.


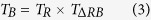


The CGH patterns for the object points located on each depth plane can be obtained just by shifting their PFPs according to the dislocated values from the center to those object points and adding them all, therefore the *R*-color CGH pattern of a 3-D scene, *I*_*R*_(*x*, *y*) can be expressed by Eq. (4)^35^


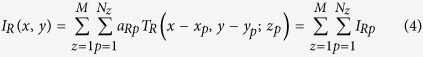


where *a*_*Rp*_, *N*_*z*_ and *I*_*Rp*_ denote the intensity value of the *R*-color object point located at (*x*_*p*_, *y*_*p*_, *z*_*p*_), the number of object points for the depth plane *z* and the *R*-color CGH pattern for the *p*^th^ object point, respectively.

The *G*-color CGH pattern can be calculated by using Eqs. (2) and (4), which is given by Eq. (5)


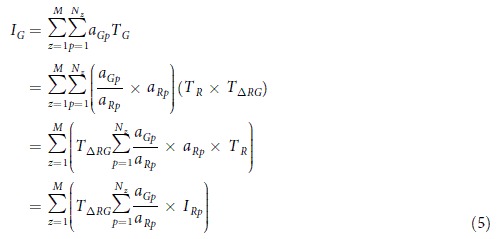


where *a*_*Gp*_ represents the intensity value of the *G*-color object point located at (*x*_*p*_, *y*_*p*_, *z*_*p*_). Equation (5) shows that the *G*-color CGH pattern for each depth plane can be calculated just by multiplying the color-compensating-PFP for the *G*-color, *T*_Δ*RG*_ to the CGH pattern generated with the *R*-color PFP. That is, color-compensation is operated on each depth plane, which means color-compensation operations can be done for all object points having the same grey level of the *R*- and G-color on the same depth at once, instead of point-by-point calculation of the *G*-color CGH pattern.

If the gray levels of the *R*- and *G*-color are same, which means *a*_*Gp*_ = *a*_*Rp*_, the *G*-color CGH pattern can be generated from that of the *R*-color just by being multiplied with the color-compensating-PFP for the *G*-color. However, in case the gray levels of the *R*- and *G*-color are different, the grey level value should be compensated before the color-compensation.

As the same manner, the *B*-color CGH pattern can be generated by using Eqs. (3) and (4), which is given by Eq. (6).


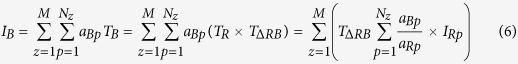


Where *a*_*Bp*_ denotes the intensity value of the *B*-color object point located at (*x*_*p*_, *y*_*p*_, *z*_*p*_). Equation (6) also tells us that the *B*-color CGH pattern can be generated from that of the *R*-color by being compensated with the color-compensating-PFP for the *B*-color, *T*_Δ*RB*_ and the difference in gray level.

Unlike the most conventional methods, where three kinds of CGH patterns for each color of the *R*, *G* and *B* need to be calculated, in the proposed method, only one-color CGH calculation and two color-compensation processes are needed if the gray levels of the object points for each of the *R*-, *G*- and *B*-color are same. Moreover, in case grey levels of the object points are same for two colors, one-color CGH calculation, two color- and one gray level- compensation processes are required. Furthermore, in case all grey levels of the object points are different, one-color CGH calculation, two color- and two gray level-compensation processes are required.

The calculation times required for color- or grey level-compensations appear to be much smaller than those for the CGH calculations, thus three-color CGH calculation time of the proposed CT-NLUT method can be much shortened than that of the conventional NLUT method.

## Additional Information

**How to cite this article**: Kim, S.-C. *et al.* Accelerated one-step generation of full-color holographic videos using a color-tunable novel-look-up-table method for holographic three-dimensional television broadcasting. *Sci. Rep.* **5**, 14056; doi: 10.1038/srep14056 (2015).

## Supplementary Material

Supplementary Information

Supplementary Information

## Figures and Tables

**Table 1:** Average number of calculated object points of the conventional and proposed methods.

**Average number of calculated object points**	**NLUT**	**CT-NLUT**			**TR/NLUT**	**TR/CT-NLUT**		
		**3-color**	**2-color**	**1-color**		**3-color**	**2-color**	**1-color**
	200,000	8,478	9,169	182,353	39,262	15,793	4,814	18,655

*3-color: the number of object points with the same gray levels for all three-color, 2-color: the number of object points with the same gray levels only for two colors, 1-color: the number of object points with different gray levels for all three colors.

**Table 2:** Average calculation time per one object-point and memory capacities of the conventional and proposed methods.

	**NLUT**	**CT-NLUT**	**TR/NLUT**	**TR/CT-NLUT**
Average calculation time per one object-point	60.67 *ms*	42.06 *ms*	12.30 *ms*	2.55 *ms*
Memory capacity	8.01 GB	27.34 KB	8.01 GB	27.34 KB

**Table 3:** Detailed compositions of the Calculation time per one object-point of the conventional and proposed methods.

		**NLUT**	**CT-NLUT**	**TR/NLUT**	**TR/CT-NLUT**
Total calculation time per one object-point		60.67 *ms* (100%)	42.06 *ms* (69.33%)	12.30 *ms* (20.27%)	2.55 *ms* (4.20%)
Detailed calculation time for	Preprocessing	−	−	0.07 *μs*	0.07 *μs*
	*R*-color CGH	20.22 *ms*	18.12 *ms*	4.10 *ms*	1.56 *ms*
	*G*- & *B*-color CGHs	40.45 *ms*	23.94 *ms*	8.19 *ms*	0.99 *ms*
