# Supplementary material for: Accelerated one-step generation of full-color holographic videos using a color-tunable novel-look-up-table method for holographic three-dimensional television broadcasting
Source: Sci Rep. 2015 Sep 11;5:14056. doi: 10.1038/srep14056 (PMC4566101; doi:10.1038/srep14056)
Supplement: Supplementary Information [file srep14056-s2.doc]

**Supplementary Information**

Accelerated one-step generation of full-color holographic videos using a color-tunable novel-look-up-table method for holographic three-dimensional television broadcasting

Seung-Cheol Kim, Xiao-Bin Dong & Eun-Soo Kim*

HoloDigilog Human Media Research Center (HoloDigilog), 3D Display Research Center (3DRC), Kwangwoon University, 447-1 Wolge-Dong, Nowon-Gu, Seoul 139-701, Korea

**1. Novel-look-up-table (NLUT) method**

Figure S1 shows an overall block-diagram of the novel-look-up-table (NLUT) method [1]. It consists of three steps: construction of the NLUT, generation of the computer-generated hologram (CGH) pattern for 3-D objects using the NLUT and reconstruction of 3-D object images. First, the NLUT is constructed only with fringe patterns of the object points located at each center of the depth-dependently sliced image planes of the objects, which are called principal-fringe-patterns (PFPs). The CGH patterns are then generated using these PFPs pre-calculated and stored in the NLUT, and from which 3-D object images are finally reconstructed.


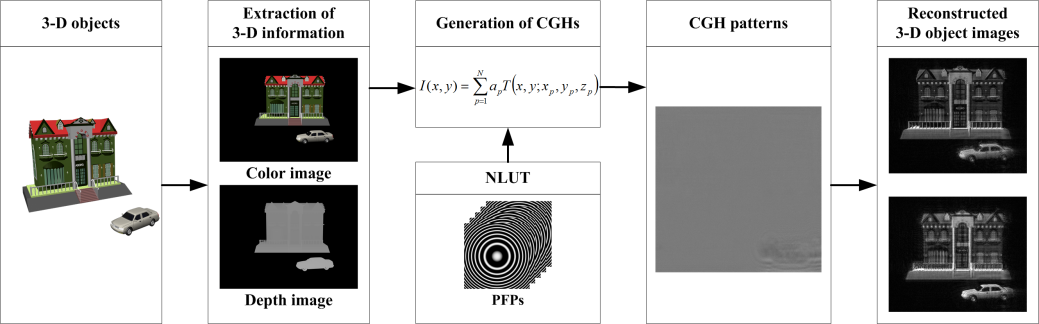


Fig. S1 Overall block-diagram of the NLUT method [1].

**1.1 Construction of the NLUT**

Basically, a 3-D object image to be generated can be approximated as a collection of self-luminous points of light. Here, horizontal, vertical and depth locations of an object point are specified as *x_p_*, *y_p_* and *z_p_*, respectively and each object point has an associated real-valued magnitude of *a_p_*. Contrary to the conventional LUT which is composed of fringe patterns for all of the object points, the NLUT consists of only the fringe patterns of the object points located at each center of the depth-dependently sliced image planes of the 3-D object, called PFPs. In other word, the NLUT contains only one PFP at each depth plane Thus, the memory capacity of the NLUT can be dramatically reduced, compared to that of the conventional LUT.

Here, each PFP can be regarded as the Fresnel-zone-pattern (FZP) calculated at each depth plane, therefore the unity magnitude PFP for the center object point (0, 0, *z_p_*) on the image plane having a depth of *z_p_*, *T*(*x*, *y*; *z_p_*) can be defined as Eq. (1).

$T(x,y;z_{p})\equiv\exp\left[j\frac{k}{z_{p}}\left\{\left(x-x_{0}\right)^{2}+\left(y-y_{0}\right)^{2}\right\}\right]$ (1)

**1.2 Calculation of CGH patterns**

Figure S2 shows a process to generate the hologram pattern for a 3-D object image with the NLUT method. As a sample object, we consider a 3-D object consisted of four points located on two kinds of depth planes: *A*(-*x*_1_, *y*_1_, *z*_1_), *B*(*x*_2_, -*y*_2_, *z*_1_), *C*(-*x*_3_, -*y*_3_, *z*_2_), and *D*(*x*_4_, *y*_4_, *z*_2_) as seen in Fig. S2(a). Of course, PFPs for the center object points on each sliced image plane are pre-calculated and stored in the NLUT.


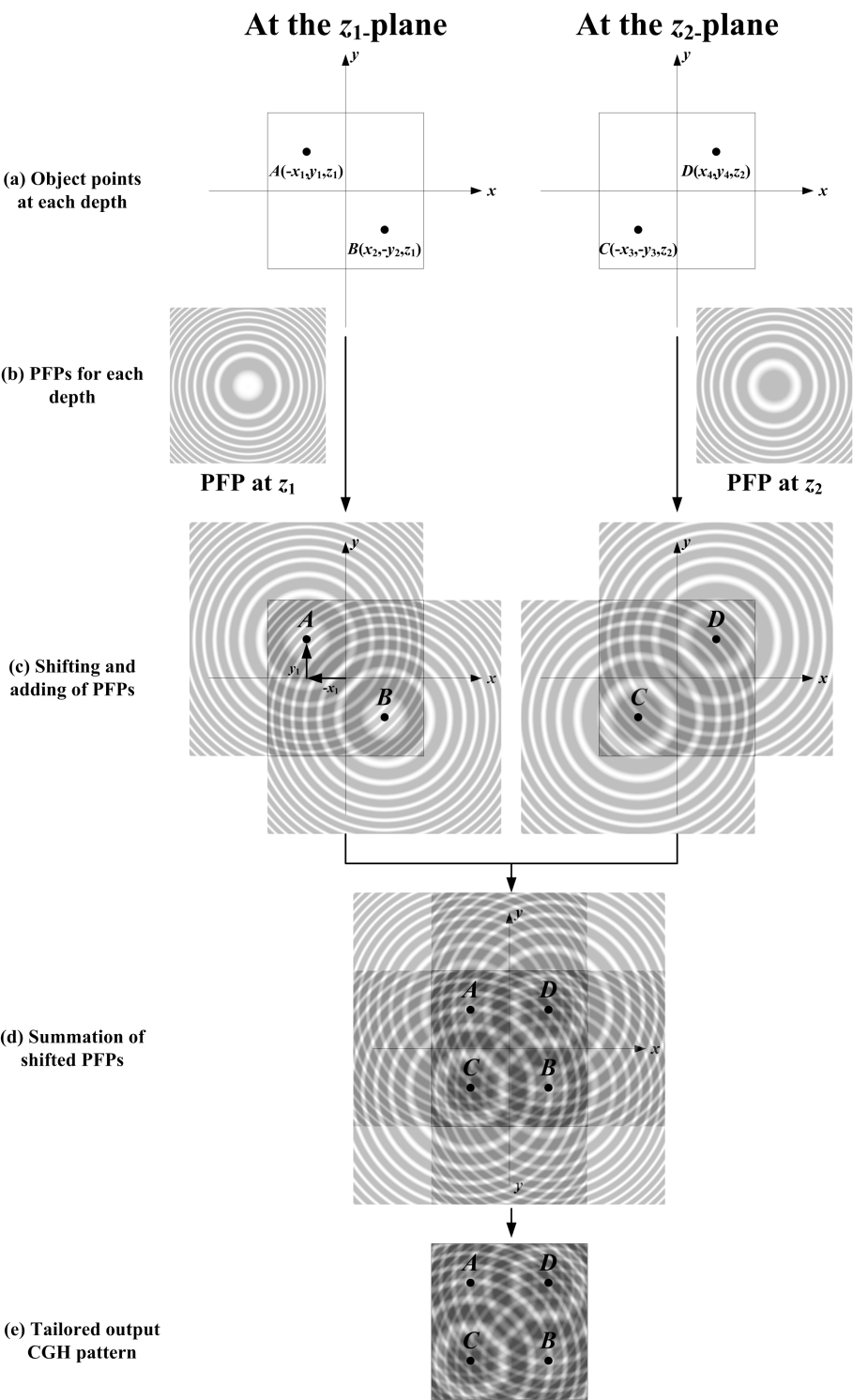


Fig. S2 Generation process of the CGH pattern using the NLUT method [1].

Basically, only two kinds of the PFPs for this sample 3-D object are stored in the NLUT. That is, one is the fringe pattern for the object point located on the center of the image plane of (0, 0, *z*_1_) and the other is the fringe pattern for the object point located on the center of the image plane of *z*_1_ as seen in Fig. S2(b). With these two PFPs, fringe patterns for four object points can be then calculated through simple shifting and adding operations. Here, the object point *A*(-*x*_1_, *y*_1_, *z*_1_) is just displaced by -*x*_1_ and *y*_1_, respectively, in the direction of *x* and *y*, from the center point *O*(0, 0, *z*_1_) on the image plane of *z*_1_. Thus, the diffraction pattern for this point can be obtained by simply shifting the pre-calculated PFP for the center point contained in the NLUT with amounts of -*x*_1_ and *y*_1_ in the direction of *x* and *y*, respectively, which is shown in Fig. S2(c). Following the same procedure, the diffraction pattern for the object point of *C*(-*x*_3_, -*y*_3_, *z*_2_) located on another image plane of *z*_2_ can be also obtained just shifting the pre-calculated PFP for the center image point *O*(0, 0, *z*_2_) stored in the NLUT with amounts of -*x*_3_ and -*y*_3_ in the direction of *x* and *y*, respectively, which is also shown in Fig. S2(c). These processes are performed for all four object points, and then its corresponding shifted versions of PFPs are obtained and added together to get the final fringe pattern for this object as seen in Fig. S2(d).

Here it is noted that because the final fringe pattern is obtained by overlapping 4 kinds of shifted versions of PFPs, its size would be increased, compared to that of the original fringe pattern of Fig. S2(d). Thus, the final CGH pattern should be tailored from this region overlapped with four shifted versions of PFPs as a pre-determined size as shown in Fig. S2(e).

Basically, the CGH pattern for the object *I*(*x*, *y*) in the NLUT method can be expressed in terms of the shifted versions of pre-calculated PFPs of Eq. (1) as shown in Eq. (2).

$I(x,y)=\sum_{p=1}^{N} a_{p}T\left(x-x_{p},y-y_{p};z_{p}\right)$ (2)

Where *N* denotes the number of object points. Equation (2) shows that the CGH pattern of an object can be obtained just by shifting the PFPs depending on the displaced values of image points from the reference points on each image plane and adding up all together. That is, in the NLUT method, the CGH pattern can be generated by multiplying the amplitudes of each object point to the corresponding PFPs, which are pre-calculated for each depth plane, and shifting them depending on the displaced values of the object points in the *x* and *y* directions, and adding them together.

**1.3 Two-step CGH calculation process of the NLUT**

Here, it must be noted that the NLUT, contrary to other methods, generates the CGH pattern of a 3-D scene based on a two-step process: pre- and main-processing [2-5]. In the pre-processing step, the number of object points to be calculated has been minimized by removing as much redundant object data between the consecutive 3-D video frames as possible by using motion estimation and compensation-based data compression algorithms. In the following main-processing step, CGH patterns only for those compressed object data obtained from the pre-processing are computed by using the NLUT based on the simple shifting and addition operations of PFPs [1]. This is the unique CGH calculation process carried out in the NLUT method by taking advantage of its shift-invariance property [2-6].

Several types of NLUTs employing the pre-processing steps for eliminating the temporal redundancy between the consecutive 3-D video frames have been proposed. They include temporal redundancy-based NLUT (TR-NLUT) [2], motion compensation-based NLUT (MC-NLUT) [3] and MPEG-based NLUT (MPEG-NLUT) [4], 3-directional motion compensation-based novel-look-up-table (3DMC-NLUT) [5], object tracking mask-based NLUT (OTM-NLUT) [6] methods.

**2. Shift-invariance property of the NLUT method**

Figure S3 shows a unique shift-invariance property of the NLUT method. As shown in Fig. S3(a), the hologram pattern for the object point *A*(0, 0, *z*_1_) represented by ‘PFP-*A*’ is recorded as a form of Fresnel-zone-plate (FZP) in the NLUT method. Here, the center of ‘PFP-*A*’ is positioned at (0, 0, 0) as shown in Fig. S3(b), so the object point *A'* reconstructed from this hologram pattern of ‘PFP-*A*’ is also located at (0, 0, *z*_1_).

Now, if this hologram pattern of ‘PFP-A’ is shifted to the location of (*x*_1_, 0, 0) along the *x*-direction, then the object point *A''* reconstructed from the shifted version of ‘PFP-*A''*’ is also moved to the new location of (*x*_1_, 0, 0). As the same manner, in case the ‘PFP-*A*’ is shifted to the location of (0, *y*_1_, 0) along the *y*-direction, then the object point *A'''* reconstructed from the shifted version of ‘PFP- *A'''*’ is also moved to the new location of (0, *y*_1_, *z*_1_).


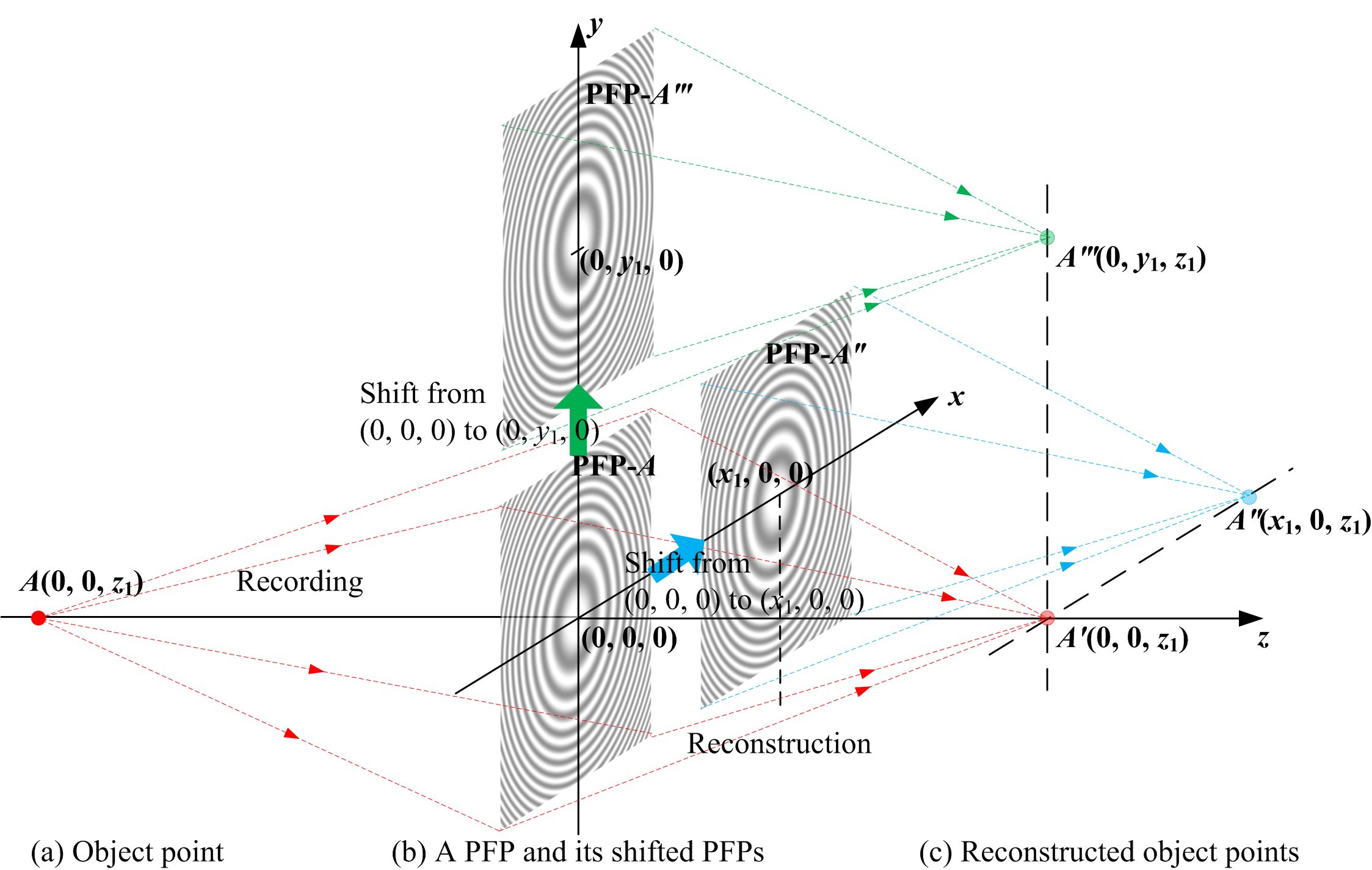


Fig. S3 Shift-invariance property of the NLUT method [3].

That is, in the NLUT method, if an object point moves, then its corresponding PFP is also shifted and the object point reconstructed from this shifted PFP is found to be at the moved location, which confirms the shift-invariance of the NLUT method. In other words, hologram patterns for the moved object points can be computed by simply shifting the corresponding PFPs with moving amounts. Based on this unique shift-invariance property of the NLUT method, various types of spatial and temporal redundancy-based NLUT methods can be realized.

Here, this unique shift-invariance property of the NLUT can be directly applied for the CGH generation of a 3-D moving object, where a 3-D object is assumed to be composed of a set of object points. As shown in Fig. S4, if the center of a 3-D object moves by *x*_1_ and *y*_1_ along the *x* or *y* directions, respectively, on a fixed object plane, the hologram patterns corresponding to each of these shifted objects can be generated by simply shifting the hologram pattern *I_A'_* (*x*, *y*) for the object located at (0, 0, *z*_1_) with amounts of *x*_1_ and *y*_1_, respectively, which are shown in Eqs. (3) and (4).

$I_{A''}(x,y)=I_{A'}(x-x_{1},y)$ (3)

$I_{A'''}(x,y)=I_{A'}(x,y-y_{1})$ (4)

Where *I_A'_* (*x*, *y*), *I_A''_* (*x*, *y*) and *I_A'''_* (*x*, *y*) represents the CGH patterns for the 3-D object at the location of *A'*, *A''* and *A'''*, respectively


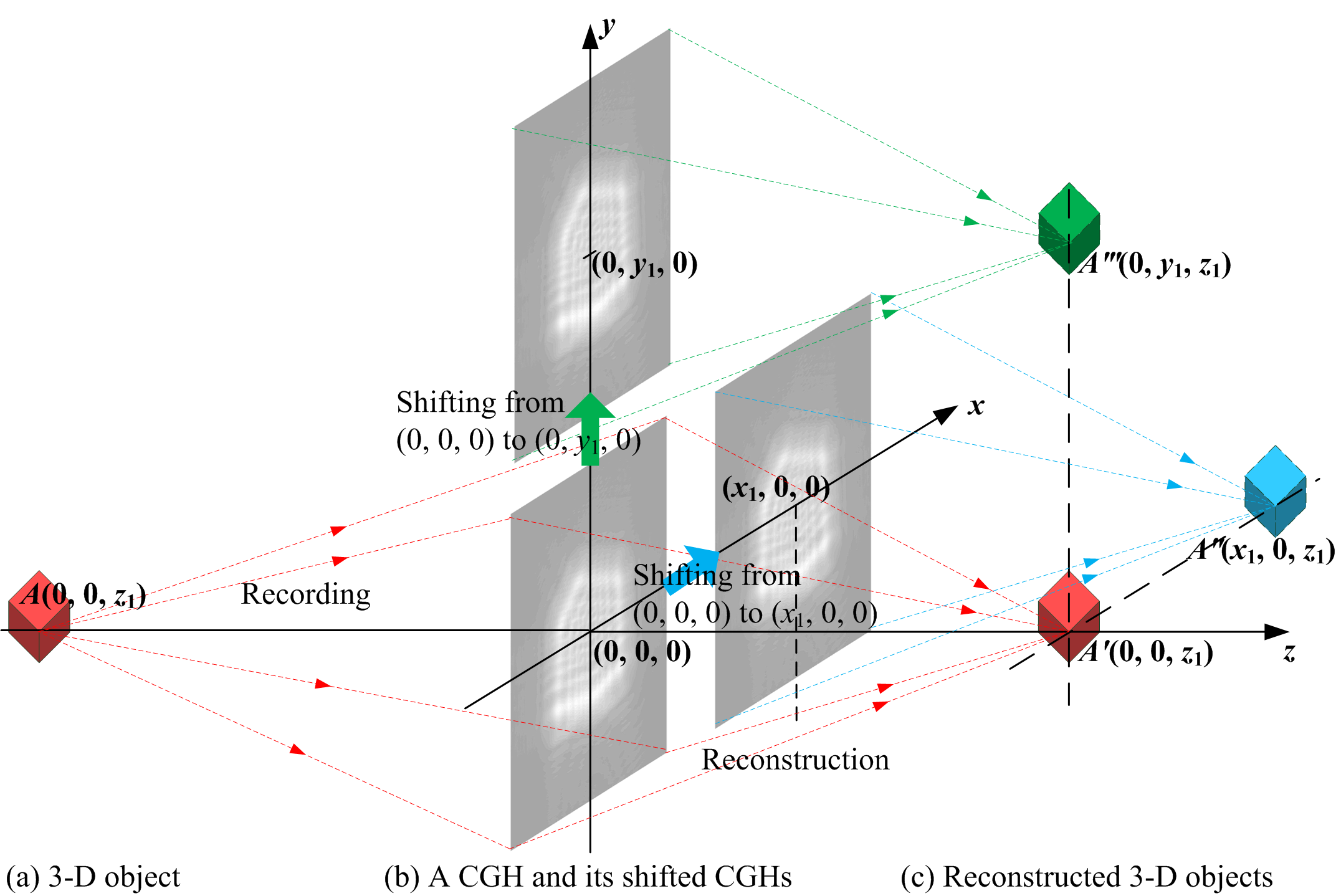


Fig. S4 CGH generation process of the moving objects based on the shift-invariance property of the NLUT [3].

Therefore, if the shifted distance of the 3-D object in an input scene is extracted, then with this value the hologram pattern for the moved 3-D scene can be generated without additional calculation of its hologram pattern by taking advantage of the shift-invariance property of the NLUT method.

**3. Thin-lens property of the NLUT**

Since the PFPs for each depth layer are calculated as forms of FZPs in the NLUT, these can be treated as thin-lenses with different focal lengths corresponding to their depth planes [7].


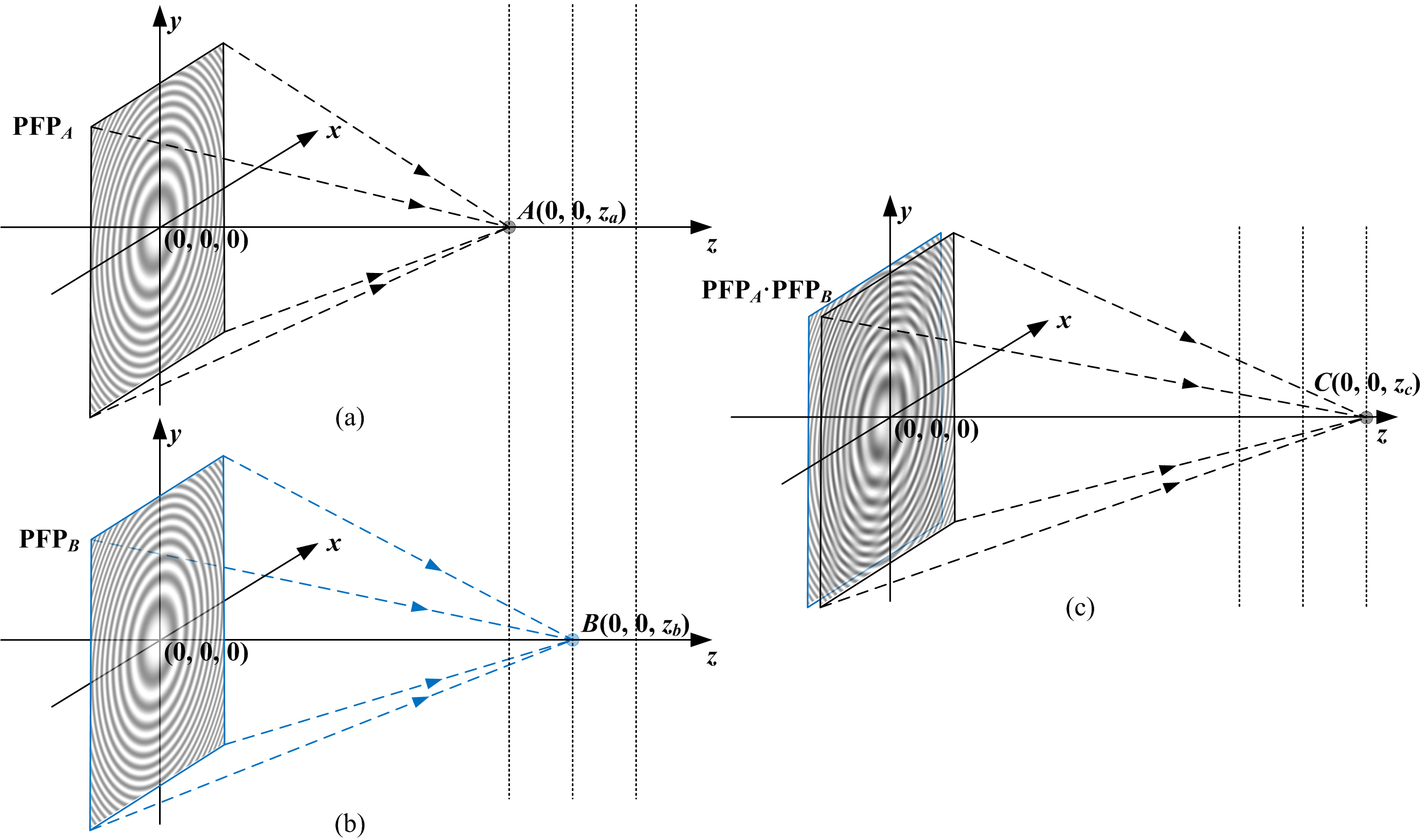


Fig. S5 Conceptual diagram for showing a thin-lens property of the PFP: (a) PFP*_A_* with the focal length of *z_a_*, (b) PFP*_B_* with the focal length of *z_b_*, (c) PFP*_C_* with the focal length of *z_c_* generated by multiplying PFP*_A_* and PFP*_B_* [7].

Figure S5 shows the conceptual diagram of a thin-lens property of the NLUT. As seen in Figs. S5(a) and S5(b), object points *A* and *B* are reconstructed with PFP*_A_* and PFP*_B_*, respectively. Thus, PFP*_A_* and PFP*_B_* with focal lengths of *z_a_* and *z_b_*, can be defined by Eqs. (5) and (6), respectively by using Eq. (1).

$T_{A}(x,y)\equiv\exp\left[j\frac{k}{z_{a}}\left\{\left(x-x_{0}\right)^{2}+\left(y-y_{0}\right)^{2}\right\}\right]$ (5)

$T_{B}(x,y)\equiv\exp\left[j\frac{k}{z_{b}}\left\{\left(x-x_{0}\right)^{2}+\left(y-y_{0}\right)^{2}\right\}\right]$ (6)

Here, if the PFP*_B_* is sandwiched with the PFP*_A_* to make a new composite PFP*_C_* as seen in Fig. S5(c), the PFP*_C_*, *T_C_*(*x*, *y*) and its focal length, *z*_c_ can be represented by Eq. (7) and (8), respectively.

$T_{C}(x,y)\equiv T_{A}(x,y)\cdot T_{B}(x,y)=\exp\left[j\frac{k}{z_{c}}\left\{\left(x-x_{0}\right)^{2}+\left(y-y_{0}\right)^{2}\right\}\right]$ (7)

$1/z_{c}=1/z_{a}+1/z_{b}$ (8)

Equation (7) shows that the PFP*_A_* with the focused depth plane of *z_a_* can be shifted to the new focused depth plane of *z_c_* just by being attached with the PFP*_B_* having the focused depth plane of *z_b_*. Here, in case the PFP having the positive focal-length such as a convex lens is attached, the focal length of the composite PFP gets decreased. On the other hand, for the PFP with the negative focal-length such as a concave lens, the corresponding focal-length of the composite PFP gets increased. Thus, with this thin-lens property of the NLUT, depth shifting of object points or a 3-D object can be possible.

**4. Achromatic thin-lens property of the NLUT**

Basically, a single lens cannot focus the input beam, which is composed of different colors, right on the same plane at the same time because the focal-length of a lens depends on its index of refraction, which differs for each color. Thus, a chromatic aberration may inevitably occur in the conventional lens system, which has been corrected by using an achromatic lens [8]. Here, an achromatic lens is fabricated to focus two colors on the same plane. The most common type of an achromatic lens is the achromatic doublet, which is composed of two individual convex and concave lenses. That is, the differences in focal-length due to different colors can be compensated just by attaching a concave lens to the convex lens.

In the NLUT, PFPs for each depth planes of a 3-D scene are calculated as forms of FZPs, thus those PFPs can be treated as thin-lenses with their own focal lengths as mentioned in Section 3. Therefore, each PFP may show a chromatic aberration just like the optical lens system. That is, in case a PFP for the red color is reconstructed with the red color, the corresponding object point is reconstructed on the predetermined depth distance. However, if this PFP is reconstructed with the green color, the depth distance of the reconstructed object point moves to a new location due to its chromatic aberration.

However, this chromatic aberration of a PFP can be corrected by combined use of the corresponding color-compensating PFP (CC-PFP) just like the achromatic doublet lens. That is, the PFP for the red color can be transformed into that for the green color just by being attached with the CC-PFP for the green color, which results in a correction of the depth distance of the reconstructed object point. In other words, just by attaching appropriate CC-PFPs to the PFP for one color, PFPs for other colors can be generated.


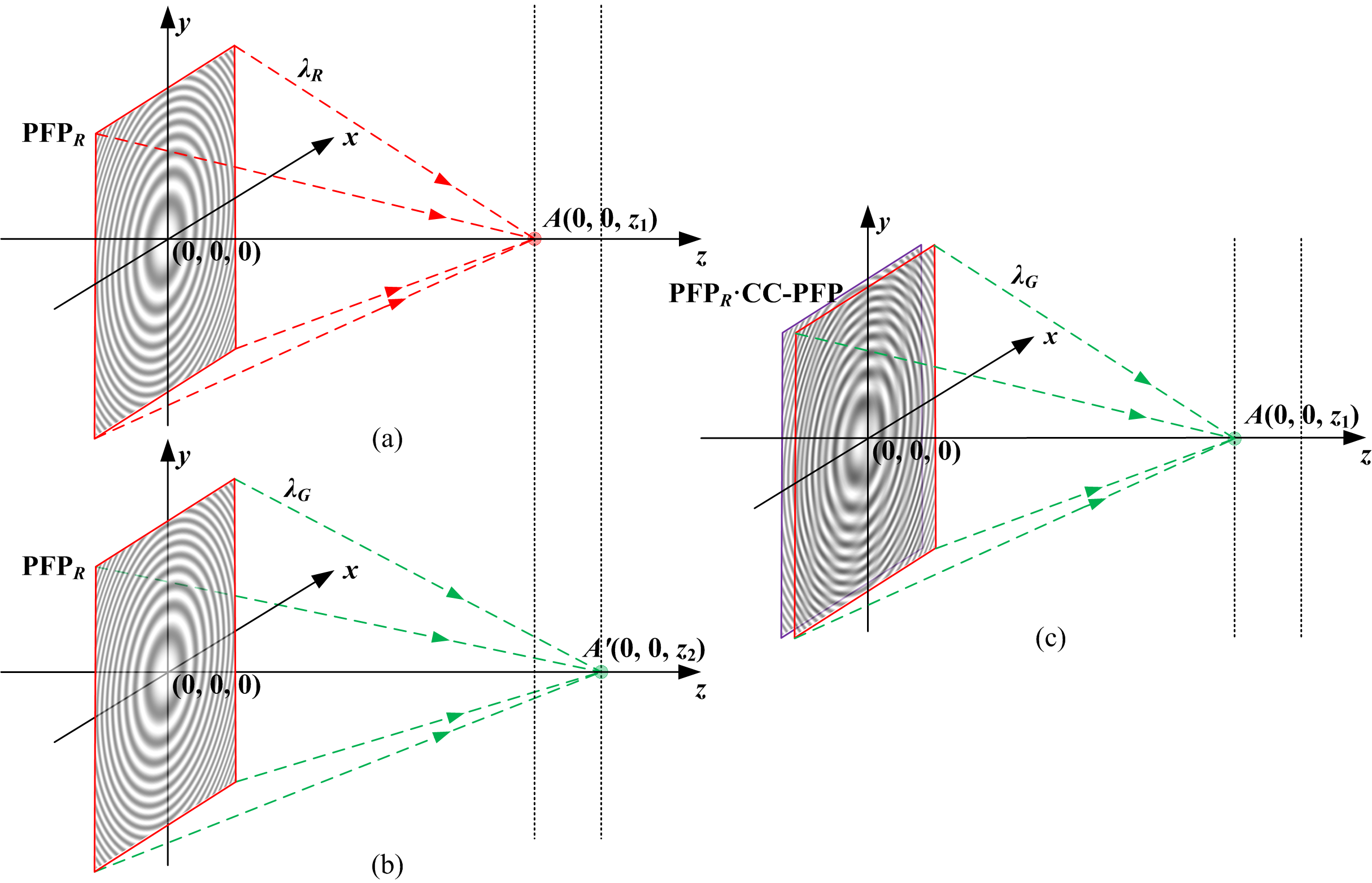


Fig. S6 Conceptual diagram for showing an achromatic lens property of the PFP: (a) Reconstruction of the PFP*_R_* with the red color, (b) Reconstruction of the PFP*_R_* with the green color: (c) Reconstruction of the PFP*_G_* with the focal length of *z*_1_ generated by multiplying the CC-PFP to the PFP*_R_*.

Figure S6 show an achromatic thin-lens property of the PFP. As seen in Fig. S6(a), an object point *A* is reconstructed with the PFP*_R_*. Thus, the PFP*_R_* of the red color with the focal length of *z*_1_ can be defined by Eq. (9) using Eq. (1).

$\begin{matrix} T_{R}(x,y)=\exp\left[j\frac{k_{R}}{2z_{1}}\left\{\left(x-x_{0}\right)^{2}+\left(y-y_{0}\right)^{2}\right\}\right]=\exp\left[j\frac{k_{R}\left(k_{G}/k_{G}\right)}{2z_{1}}\left\{\left(x-x_{0}\right)^{2}+\left(y-y_{0}\right)^{2}\right\}\right] \\ \begin{matrix} & & =\exp\left[j\frac{k_{G}\left(k_{R}/k_{G}\right)}{2z_{1}}\left\{\left(x-x_{0}\right)^{2}+\left(y-y_{0}\right)^{2}\right\}\right] \end{matrix}=\exp\left[j\frac{k_{G}}{2z_{2}}\left\{\left(x-x_{0}\right)^{2}+\left(y-y_{0}\right)^{2}\right\}\right] \end{matrix}$ (9)

$z_{2}=\frac{k_{G}}{k_{R}}z_{1}$ (10)

Where *k_R_* and *k_G_*, respectively, represent the wave numbers for the red and green colors. Here, if the PFP*_R_* is reconstructed with the wavelength of the red color, the object point is reconstructed at the distance of *z*_1_. However, if the PFP*_R_* is reconstructed with the wavelength of the green color as shown in Fig. S6(b), the object point is reconstructed at the distance of *z*_2_ as shown in Eqs. (9) and (10). That is, the distance of the reconstructed object point moves to a new location due to the chromatic aberration. Therefore, the distance of the reconstructed object point can be compensated by multiplying the CC-PFP to the PFP*_R_* just like an achromatic doublet lens as shown in Eqs. (11) and (12).

$\begin{matrix} T_{G}(x,y)=\exp\left[j\frac{k_{R}}{2z_{1}}\left\{\left(x-x_{0}\right)^{2}+\left(y-y_{0}\right)^{2}\right\}\right]\exp\left[j\frac{k_{\Delta RG}}{2z_{1}}\left\{\left(x-x_{0}\right)^{2}+\left(y-y_{0}\right)^{2}\right\}\right] \\ \begin{matrix} & & =\exp\left[j\frac{k_{R}+k_{\Delta RG}}{2z_{1}}\left\{\left(x-x_{0}\right)^{2}+\left(y-y_{0}\right)^{2}\right\}\right] \end{matrix}=\exp\left[j\frac{k_{G}}{2z_{1}}\left\{\left(x-x_{0}\right)^{2}+\left(y-y_{0}\right)^{2}\right\}\right] \end{matrix}$ (11)

$k_{\Delta RG}=k_{G}-k_{R}$ (12)

Where *k_ΔRG_* means the wave number of the CC-PFP. Therefore, green and blue-color PFPs can be generated from the red-color PFP simply by being multiplied with their corresponding CC-PFPs having difference wavelengths between the red and each of the green and blue colors.

**5. Temporal redundancy-based NLUT (TR-NLUT) method**

Contrary to the 2-D video, 3-D video is a collection of sequential 3-D images having depth data as well as intensity. Just like the case of the 2-D video images, neighboring moving pictures in the 3-D video also differ slightly from each other, but in the 3-D video images intensity and depth data are simultaneously changed.


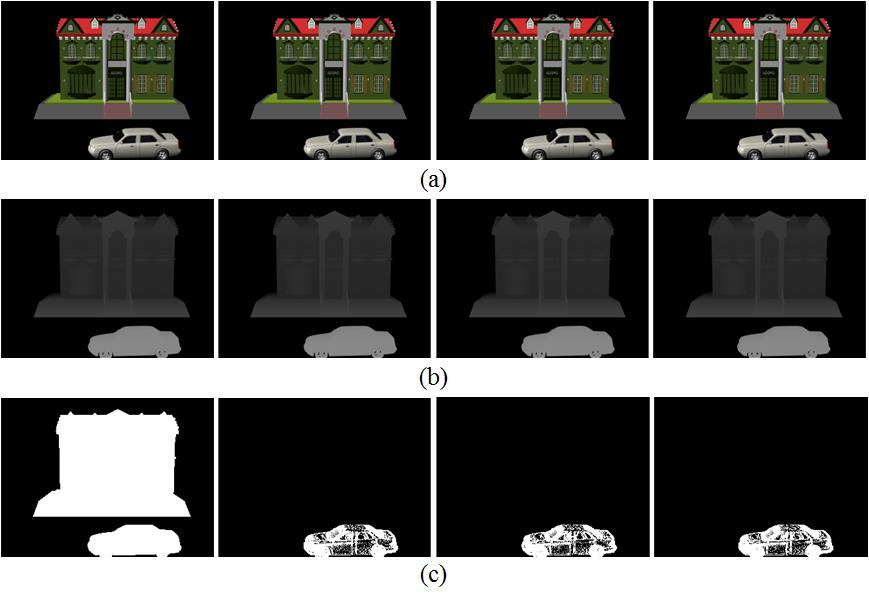


Fig. S7 Adjacent four frames of a 3-D video: (a) Intensity images, (b) Depth images, (c) difference data between current frame and previous frame [2].

For example, Fig. S7 shows the adjacent four frames of the 3-D video images composed of a fixed house and a moving car. In these four frames, only the car is moving and the other part of the image is not changed. Thus, there exist slight changes in intensity and depth between the consecutive frames. The difference data between previous frame and current frame is shown in Fig.S7(c), the same part with the previous frame doesn’t need to be calculated again, in other words, only the different part with previous frame needs to be calculated and added to previous frame hologram to get the hologram of current frame. The number of calculated object points is reduced dramatically compared with the conventional NLUT method which doesn’t consider the temporal redundancy of the 3D video. As a result, the calculation time for generation of the 3-D video images can be dramatically shortened.

Accordingly, temporal redundancy-based NLUT (TR-NLUT) method has been proposed for fast generation of CGHs of 3D video objects. The CGH of the first frame is calculated point by point using conventional NLUT method. And for the following frames, the intensity and depth data of the frame under calculation are extracted and compared to those of the previous frame, the difference area between current frame and previous frame can be extracted. The CGH of the difference area on previous frame and the CGH of the difference area on current frame will be calculated, subtracted and added to the CGH of the whole previous frame to get the CGH of current frame, as shown in Eq. (13).

$I_{n}(x,y)=I_{n-1}(x,y)-\sum_{p=1}^{N_{d}} a_{p_{n-1}}U_{n-1}(x-x_{p},y-y_{p};z_{p})+\sum_{p=1}^{N_{d}} a_{p_{n}}U_{n}(x-x_{p},y-y_{p};z_{p})$ (13)

Where *I_n_* is the CGH pattern for the *n-th* frame, *N_d_* is the number of different image points in 3-D data between the *n*-*th* and (*n*-1)-*th* frames. Moreover, *U_n_*(*x*, *y*; *z_p_*) means the PFPs of the *n*-*th* frame as shown in Eq. (14).

$U_{n}(x,y;z_{p})=\left\{\begin{matrix} T(x,y;z_{p}) \\ 0 \end{matrix}\right.\begin{matrix} & & & & \\ & & & & \end{matrix}\begin{matrix} for \\ for \end{matrix}\begin{matrix} \\ \end{matrix}\begin{matrix} changed\begin{matrix} \end{matrix}\begin{matrix} part \end{matrix} \\ unchanged\begin{matrix} \end{matrix}\begin{matrix} part \end{matrix} \end{matrix}$ (14)

And then, the calculated CGH pattern of the second frame *I_n_*(*x*, *y*) is moved to the CGH video output as well as stored in the previous frame buffer of the CGH. These processes are repeated for all of the video frames.

Therefore, TR-NLUT can be applied to proposed CT-NLUT method. That is, object points to be calculated in 3-D video frame can be reduced and calculation time can be reduced by combined use of TR-NLUT and CT-NLUT methods.

**6. Compressed-NLUT method (C-NLUT)**

The computational speed of the NLUT can be further enhanced by decreasing the CGH calculation time for those compressed object data obtained from the pre-processing of the first stage. However, the conventional NLUT methods, which employ pre-processing schemes, must require two-step calculation processes for each 3-D video frame, which actually limits the computational speed [2]. However, this two-step CGH calculation process limits the computational speed of the conventional NLUT methods.

To solve this problem, a new type of the NLUT, which is so-called compressed novel-look-up-table (C-NLUT), to fast calculate the CGH patterns of 3-D video frames with only one-step process based on its unique thin-lens property, is proposed. The C-NLUT is composed of only two PFPs, one is the baseline PFP (B-PFP) designated for the 1^st^ depth layer of the 3-D video frame and the other is the depth-compensating PFP (DC-PFP) for compensating the depth differences between the baseline and other depth layers.

Basically, in the NLUT method, the PFPs for each depth layer are calculated as forms of Fresnel zone plates (FZPs), thus these can be treated as thin-lenses with different focal lengths corresponding to their depth layers. Therefore, based on this thin-lens property, hologram patterns for each depth layer of a 3-D video frame can be generated by calculating the hologram patterns for all depth layers only by using the B-PFP, and then transforming them into those for their depth layers just by being multiplied with corresponding DC-PFPs, which act just like thin-lenses having their focal lengths corresponding to depth differences between the baseline and other depth layers. In other words, the proposed method can calculate the CGH patterns of 3-D video frames just by one-step multiplication process instead of two-step subtraction and adding operations in the conventional methods, which results in a great acceleration of the computational speed of the NLUT.

Figure S8 shows an overall block-diagram of the compressed novel-look-up-table (C-NLUT) method, which is largely composed of three stages [7]. At the first stage, intensity and depth differences between two consecutive (previous and current) frames of input 3-D video images are extracted. At the second stage, the CGH pattern for the first 3-D video frame is calculated by combined use of baseline PFP (B-PFP) and depth-compensating-PFP (DC-PFP). That is, the hologram patterns for the object points located on each depth layer of the first 3-D video frame are calculated only by using the B-PFP, which is designated to the PFP for the 1^st^ depth layer, and they are then transformed into their original depth layers by being multiplied with respective DC-PFPs. The CGH pattern for the first 3-D video frame can be finally obtained by accumulating all those hologram patterns calculated for each depth layer.


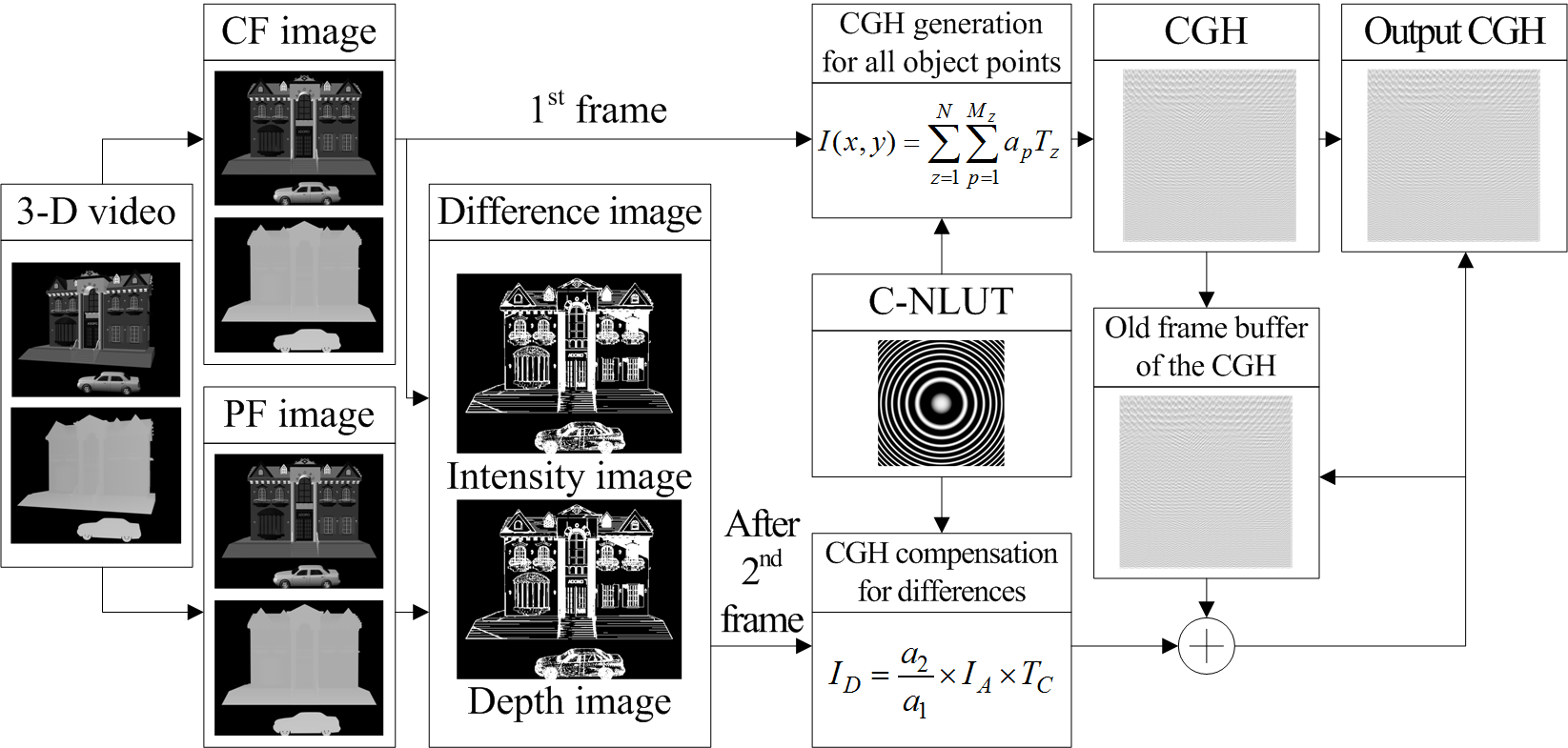


Fig. S8 Overall block-diagram of the C-NLUT method to generate video holograms of a 3-D scene by combined use of B-PFP and DC-PFP (PF: previous frame, CF: current frame) [7].

Moreover, for the remaining 3-D video frames, only the hologram patterns for the changed object points in both previous and current video frames are compensated. That is, intensity and depth changes of the object points can be compensated just by multiplying their intensity differences and corresponding DC-PFPs, respectively, to the hologram patterns for the changed object points. In the third stage, the calculated CGH patterns for each video frame are transmitted to the CGH video output as well as stored in the previous frame buffer of the CGH for computing the CGH pattern for the next 3-D video frame.

In the prosed method, R-color CGH is generated by using conventional method because R-color CGH is the reference hologram pattern for color compensation. Therefore, C-NLUT is used to generate for R-color CGH.

**Reference**

1. Kim, S.-C. & Kim, E.-S. Effective generation of digital holograms of 3-D objects using a novel look-up table method. *Appl. Opt.* **47**, D55-D62 (2008).
2. Kim, S.-C., Yoon, J.-H. & Kim, E.-S. Fast generation of 3-D video holograms by combined use of data compression and look-up table techniques. *Appl. Opt.* **47**, 5986-5995 (2008).
3. Kim, S.-C., Dong, X.-B., Kwon, M.-W. & Kim, E.-S. Fast generation of video holograms of three-dimensional moving objects using a motion compensation-based novel look-up table. *Opt. Express* **21**, 11568-11584 (2013).
4. Dong, X.-B., Kim, S.-C. & Kim, E.-S. MPEG-based novel-look-up-table method for accelerated computation of digital video holograms of three-dimensional objects in motion. *Opt. Express* **22**, 8047-8067 (2014).
5. Dong, X.-B., Kim, S.-C. & Kim, E.-S. Three-directional motion compensation-based novel-look-up-table for video hologram generation of three-dimensional objects freely maneuvering in space. *Opt. Express* **22**, 16925-16944 (2014).
6. Kwon, M.-W., Kim, S.-C., Ho, Y.-S. & Kim, E.-S. Object tracking mask-based NLUT on GPUs for real-time generation of holographic videos of three-dimensional scenes. *Opt. Express* **23**, 2101-2120 (2015).
7. Kim, S.-C. & Kim, E.-S. Fast one-step calculation of holographic videos of three-dimensional scenes by combined use of baseline and depth-compensating principal fringe patterns. *Opt. Express* **22**, 22513-22527 (2014).
8. B. E. A. Saleh and M. C. Teich, *Fundamentals of Photonics* (Wiley, 2nd edition, 2007).
